# Impact of rye-based evening meals on cognitive functions, mood and cardiometabolic risk factors: a randomized controlled study in healthy middle-aged subjects

**DOI:** 10.1186/s12937-018-0412-4

**Published:** 2018-11-06

**Authors:** Jonna C. Sandberg, Inger M. E. Björck, Anne C. Nilsson

**Affiliations:** 1 Department of Food Technology, Engineering and Nutrition, Lund University, P.O. Box 124, SE-221 00 Lund, Sweden; 2 Food for Health Science Centre, Lund University, P.O. Box 124, SE-221 00 Lund, Sweden

**Keywords:** Whole grain rye, Dietary fiber, Dietary prevention, Type 2 diabetes, Obesity, Mood, Glucose regulation, Cognition, Gut hormones, Gut fermentation

## Abstract

**Background:**

Whole grain (WG) intake is associated with reduced risk of obesity, type 2 diabetes and cardiovascular disease, whereas type 2 diabetes increases the risk of cognitive decline and dementia. The purpose of this study was to investigate the effects of short-term intervention with WG rye on cognitive functions, mood and cardiometabolic risk markers in middle-aged test subjects.

**Method:**

Rye-based breads were provided to 38 healthy test subjects (aged 52-70y) during three consecutive days in a crossover study design, using white wheat flour bread (WWB) as a reference. The rye-based bread consisted of a WG rye kernel/flour mixture (1:1 ratio) supplemented with resistant starch type 2 (RS2) (RB + RS2). The last bread portion was ingested at 2100 h, and cognitive function, mood and cardiometabolic risk markers were determined the following morning, 11 − 14 h post intake.

**Results:**

In comparison to WWB, the RB + RS2 product increased ratings of mood parameters (valance, *P* < 0.001; activation *P* < 0.05). No differences were seen in the cognitive tests depending on intervention (*P* > 0.05). RB + RS2 increased insulin sensitivity (*P* < 0.05), fasting levels of gut hormones (PYY, P < 0.05; GLP-2, *P* < 0.01) and fasting concentrations of plasma acetate, butyrate and total SCFA (*P* < 0.001). In contrast, fasting levels of IL − 1β were decreased (*P* < 0.05). Insulin sensitivity was positively correlated with working memory test performance (P < 0.05).

**Conclusions:**

This study display novel findings regarding effects of WG rye products on mood, and glucose and appetite regulation in middle-aged subjects, indicating anti-diabetic properties of WG rye. The beneficial effects are suggested to be mediated through gut fermentation of dietary fiber in the RB + RS2 product.

**Trial registration:**

The study was retrospectively registered at ClinicalTrials.gov, register number NCT03275948. Registered September 8 2017.

## Introduction

The prevalence of obesity is rapidly increasing worldwide. Obesity is associated with several health consequences including increased risk of developing cardiovascular disease (CVD) and type 2 diabetes (T2D) [1]. Obesity and T2D increase the risk of developing cognitive decline and dementia [2], where Alzheimer’s disease is the most common type of dementia in diabetes patients [3]. Measures for primary prevention based on diet are thus urgently needed. Intake of dietary fiber (DF), in particular in the form of whole grain (WG), have been shown to reduce risk of obesity [4], T2D and CVD [5]. Furthermore, specific diets, e.g. diets rich in nuts, berries, fish, beans, olive oil and WG are associated with slower rate of cognitive decline in both observational [6] and in intervention studies [7]. Prospective studies further revealed that DF from cereals (primarily from WG breads and rolled oats) were positively correlated with “successful” ageing including aspects such as good cognitive function and absence of depressive syndrome [8].

Intervention studies evaluating the effects of WG foods on cognitive functions in healthy individuals are limited. However, short-term intervention studies with WG foods, such as WG barley or rye products rich in intact kernels, and hence rich in DF in the form of both non-starch polysaccharides (NSP) and resistant starch (RS), have shown anti-diabetic and anti-obesogenic potential in healthy middle-aged subjects and/or in healthy young adults [9–11]. In addition, intake of WG rye bread composed of 1:1 ratio of kernels and flour, containing also RS type 2 (RS2) from high-amylose maize (HAMRS2), resulted in decreased glucose and insulin responses in young adults the following morning after a standardized breakfast [12]. HAMRS2 was added to the bread recipe to compensate for the loss of RS (and hence also total DF) when milling the kernels to flour, and to further increase the RS above the content in intact kernels. The results support anti-diabetic potential of WG rye, and may indicate an importance of RS in the cereals to achieve metabolic benefits. The WG rye with added HAMRS2 also increased the satiety inducing gut hormone PYY and decreased free fatty acid (FFA) levels, compared to white wheat based bread (WWB). The observed benefits on glucose regulation and appetite variables were proposed to originate from colonic fermentation of the specific DF present [9, 10, 12], possible through mechanisms involving production of fermentation metabolites such as short chain fatty acids (SCFA, mainly acetic, propionic and butyric acid). It has been proposed that SCFA may stimulate the release of gut hormones such as GLP − 1 and PYY [13]; hormones which are important for appetite- and metabolic regulation [14, 15].

Brain-derived neurothophic factor (BDNF) is a neurotrophin suggested to be a potential biomarker for neuronal integrity, and is mainly located in areas of the brain that are associated with cognitive control, e.g. memory formation [16], and energy homeostasis [17]. BDNF affects e.g. appetite suppression [18] and glucose metabolism [19]. Reduced levels of BDNF have been detected in patients suffering from e.g. Alzheimer’s disease and depression [16]. BDNF is also widely distributed in the gastrointestinal tract [20]. Previously, increased plasma BDNF concentrations were observed in young healthy adults after intervention with WG rye kernel-based bread [21]. In animals (rats), prebiotic feeding (fructo-oligosaccharides and galacto-oligosaccharides) was shown to increase the expression of BDNF [22]. The effect was suggested to be mediated through a prebiotic induced increase in gut hormones, indicating involvement of colonic fermentation in regulation of BDNF. Furthermore, in mouse models administration of sodium butyrate was shown to upregulate BDNF levels in the frontal cortex [23].

Diets or foods that induce improvements of caradiometabolic risk factors may be beneficial also with regard to cognitive functions and mood. E.g., in healthy middle aged subjects it was shown that daily supplementation with n-3 PUFA for 5 weeks resulted in decreased plasma triacylglycerides and lowered systolic blood pressure, in parallel with improved performance in a working memory (WM) test [24]. Similarly, 5 weeks daily intake of a mixed berry beverage high in polyphenols, improved performed in a WM test compared to a carbohydrate matched control beverage [25]. Concomitantly the berry beverage reduced total- and LDL cholesterol, and lowered postprandial insulin concentrations. Further, in healthy subjects a bread product with the capacity to induce a metabolically favorable postprandial blood glucose profile resulted in an improved performance in a selective attention (SA) test, in comparison with intake of a high glycaemic index bread [26]. Interestingly, glucose tolerance was positively related to performance in the cognitive tests. Consequently, there seems to be a relation between cardiometabolic risk markers and cognitive functions not only in T2D subjects but also in healthy individuals. In addition it has been suggested that gut hormones may be involved in regulating cognition and mood. Thus, it has been shown that PYY may have anti-depressant effects [27] and that GLP − 1 may contribute to improve cognitive functions such as memory and learning [28].

As described above, WG rye products have the potential to improve measures of metabolic regulation in healthy subjects, and increase gut hormones such as PYY and GLP − 1 [9, 12]. However, the effects on cognitive functions and mood of short-term intervention with WG rye in healthy middle-aged subjects have not previously been studied. The aim of this study was to investigate effects of short-term intervention with WG rye bread containing a mixture of kernels and flour (1:1 ratio) supplemented with HAMRS2 (Hi-Maize®), on measures of cognitive functions (WM and SA) and mood in a cohort of healthy middle-aged subjects. In addition cardiometabolic risk markers, BDNF, gut hormones and subjective appetite were measured. Gut microbiota fermentation metabolites (SCFA and breath hydrogen (H_2_)) were determined to assess the involvement of gut fermentation. In this crossover study, WG rye bread and white wheat bread (WWB, reference) was consumed during 3 consecutive days in separate periods, before determination of test variables after a standardized breakfast at day 4.

## Material and methods

### Ethical statement

Registration of the study was made at ClinicalTrials.gov (NCT03275948). The study was approved by the Regional Ethical Board in Lund, Sweden (Reference 2016/487).

### Test subjects

A total of 43 healthy middle-aged persons were recruited to the study, 33 women and 10 men, aged 52–70 years (mean ± SD: 63.6 ± 5.3 years) and normal/slightly overweight (BMI 24.6 ± 2.7 kg/m^2^). Recruitment was performed through advertisement in local newspapers. The inclusion criteria were age between 50 and 70 years, BMI between 19 and 28 kg/m^2^, blood glucose < 6.1 mmol/L, non-smoker, no known metabolic diseases or gastro-intestinal disorders, and no known cognitive decline. At the time of enrollment, the subjects were screened for fasting (f-) blood glucose concentrations and blood pressure. For details regarding physiological test parameters at screening of the study population that completed the study, Table 2 in the Results section. High blood pressure medication was allowed but had to be kept consistent during the entire study. Intake of probiotics or antibiotics was not allowed within 4 weeks prior to the study or during the study. The subjects were required to be fluent in Swedish due to the construction of the cognitive tests. Prior to inclusion, each subject was given a full explanation, both written and oral, regarding the purpose and procedure of the study, and written informed consent was obtained from each subject. All subjects were aware of the option to withdraw from the study at any time they desired. The recruitment process started in October 2016. Trials were ongoing between November 2016 and February 2017.

### Test product and reference product

The study included a rye-based test product, a bread (rye bread: RB), composed of rye kernels and WG rye flour (ratio 1:1, cereal dry matter (dm)) supplemented with RS2 (RB + RS2). White wheat flour-based bread was included in the study as a reference product (WWB). Each test and reference product was consumed for three consecutive days as part of their habitual diet. The daily portion size was based on 75 g available carbohydrates. The breads were produced in a bakery (Inspira, Lund, Sweden).

#### RB + RS2

The rye-based test bread contained 43% rye kernels, 43% WG rye flour, and 14% added HAMRS2 flour (% dm, Hi-Maize® 260, including 60% RS2 and 40% digestible starch). The rye kernels (300 g) were boiled for 15 min in 250 g water, containing 6 g NaCl. All water was absorbed into the kernels when cooked. After 30 min of cooling; 300 g WG rye flour, 100 g Hi-Maize® 260, 6 g yeast (Jästbolaget AB, Sollentuna, Sweden) and 360 g lukewarm water were added to the kernels and mixed for 6 min in a food processor. The dough was proofed for 20 min and baked at 180 °C for 60 min. Then the bread was wrapped in a moist towel and cooled for 3 h before it was placed in a plastic bag over night. The bread was cut into portions the next morning, wrapped in aluminum foil and placed in a plastic bag. The bread portions were stored in a freezer (− 20 °C) prior to distribution. The separate commercial blends of rye flour and rye kernels were generously provided by Finax AB (Helsingborg, Sweden), and Hi-Maize® 260 was kindly provided by Kåkå (Lomma, Sweden).

#### WWB

The reference product was a 100% dm white wheat flour based bread. The white wheat flour (540 g) was mixed with 360 g lukewarm water, 4.8 g yeast and 4.8 g NaCl in a food processor for 6 min. The dough rested for 30 min and proofed for 35 min. The bread was baked at 200 °C for 40 min. Then the bread was wrapped in a dry towel and cooled for 2 h before it was cut into portions, wrapped in aluminum foil and placed in a plastic bag. The product was stored in a freezer (− 20 °C) prior to distribution. The commercial blend of white wheat flour was obtained from Kungsören AB (Järna, Sweden).

### Standardized breakfast

The standardized breakfast was served in the morning at the experimental day and consisted of 113.9 g of WWB (corresponding to 50 g available carbohydrates) without the crust. The WWB included in the standardized breakfast was baked according to the same procedure as the reference product (WWB) that is described above. The bread was served together with 200 ml water.

### Chemical analysis of test- and reference product

The test- and reference product were characterized based on total starch [29], RS [30] and NSP (soluble and insoluble) [31]. Prior to analysis of total starch and NSP, the samples were air dried and milled (IKA A11 basic mixer model A11 B, Germany). The analysis of RS in the products was performed as eaten, i.e. no prior air drying or milling. The available starch content in RB + RS2 was based on the difference between total starch and RS, whereas the available starch in the WWB was determined according to Holm et al. (1986) [32], Table 1.

**Table 1:** Characterization of test- and reference product concerning starch (total, available and resistant) and NSP^a^

Products	Portion size^b^	Total starch	Available starch^c^	RS	Insoluble DF	Soluble DF	Total DF
% dry matter							
RB + RS2	–	63.0	52.9	10.1	14.8	4.0	28.9
WWB	–	73.8	73.3	–	3.8	1.2	5.0
g/day							
RB + RS2	239.2	89.3	75.0	14.3	20.9	5.7	40.9
WWB	170.9	75.6	75.0	–	3.9	1.2	5.1

Available starch in WWB was determined according to Holm et al. (1986) [32]. Values of total and available starch are based on means of 2 replicates, RS means of 6 replicates, DF means of 3 replicates. (−) indicates that no analysis has been performed or data not available

*DF* Dietary fiber, *NSP* Non-starch polysaccharides, *RS* Resistant starch, *RB + RS2* 1:1 ratio rye flour and kernel based bread with added RS2 (14%, dm), *WWB* White wheat bread

^a^Data are presented as means

^b^Daily portion size

^c^Available starch in RB + RS2 was obtained by calculating the difference between total starch and RS

### Study design and protocol

The study had a crossover, randomized design. Impact on cognitive performance, mood and cardiometabolic risk markers was determined the following morning in a fasted and postprandial state following a standardized breakfast, after three consecutive days of intake of RB + RS2. The results on test variables after the RB + RS2 intervention were compared to the results after the WWB reference intervention. Out of the 38 subjects that completed both interventions, 19 (16 women and 3 men) started with the RB + RS2 and 19 (14 women and 5 men) started with WWB. The test subjects were instructed to standardize their meal patterns, and to avoid food rich in DF and alcohol during the intervention periods. In order to facilitate the standardization of food intake during the two intervention periods, the subjects were asked to record their diet and meal patterns. The notes were used by the subjects with the purpose to facilitate to repeat the pattern of food intake from the first to the second period. They were also instructed to avoid physical exercise the day before an experimental day and to avoid consumption of antibiotics and probiotics for 1 month prior to start of study and during the study.

The 3 day intervention periods were separated with at least 3 weeks of washout period. The daily amount of the test and reference products was divided into three portions. During the two first days, the intake of the three daily portions could be divided freely during the day to fit the test subjects’ habitual diet. On the third day of each intervention period, the subjects were instructed to consume two smaller portions (total amount of 25 g available starch) at breakfast and lunch, respectively. The last portion on the third day (based on 50 g available starch) was consumed at 0900 pm and the choice of drinks (individually standardized) was limited to water, tea or coffee (no milk or sugar). Thereafter, the subjects were fasting until the standardized breakfast the following morning. The test subjects arrived to the research unit (Food for Health Science Centre, Lund University) at 0745 am. After resting for 10 min in a sitting position the fasting blood samples were collected via venous and capillary sampling. In addition, subjective appetite ratings, subjective mood ratings and breath H_2_ excretion were measured in the fasting state. Once the fasting samples had been collected, the standardized breakfast was served (WWB and 200 ml water). The subjects were instructed to consume the breakfast within 10 to 12 min. Collection of physiological test markers and cognitive tests were performed repeatedly during 3 h. The cognitive tests started 30 min after commencing the standardized breakfast.

### Physiological test parameters

Fasting and postprandial measurements of physiological test variables were determined at both visits. Capillary blood via finger-prick was collected for determination of blood glucose (HemoCue®B-glucose, HemoCue AB, Ängelholm, Sweden) and serum (s-) insulin. Blood glucose and insulin were determined at fasting and every 15 min during the first hour, and thereafter every half hour until 180 min after commencing the standardized breakfast. Venous blood samples were collected at fasting to determine FFA and triglycerides in serum, and BDNF, CRP, GLP-1, PYY, GLP-2, IL-1β, IL-6, IL-18, lipopolysaccharide-binding protein (LBP), and SCFA in plasma. Blood collecting tubes used for analysis of GLP-1, PYY and GLP-2 contained an inhibition cocktail consisting of DPPIV-inhibitor (10 μl/ml blood) (Millipore, St Charles, USA) and aprotinin (50 μl/ml blood) (Sigma-Aldrich, St Louis, USA). Breath H_2_ excretion was measured at fasting using an EC 60 gastrolyzer (Bedfont EC60 Gastrolyzer, Rochester, England) as a course indicator of gut fermentation activity. Subjective appetite sensations (satiety, hunger and desire to eat) were measured at fasting and every half hour until 180 min. Registration of subjective mood ratings (valence and activity) were made at fasting and every hour until 180 min. Subjective appetite and mood ratings were registered using sheets of 100 mm Visual Analogue Scale (VAS). The ratings were assessed using separate scales for each test variable and each test point. The test was composed of black scales (100 mm lines) on a white A4 paper. Each line (scale) was marked in the middle (at 50 mm) to facilitate a correct response. The subjects were instructed that the mark in the middle indicate an answer corresponding to not agree in any direction regarding the subjective rating of the test variable. They were instructed that the more deviation the judgement was marked on the scale from the middle mark, the stronger judgement of the test variable. One end of the scale (0 mm) corresponding to “no agreement at all” regarding the subjective test variable, and the other end of the scale (100 mm) correspond to a total (100%) agreement with the subjective test variable. Determination of serum insulin concentrations was performed using a solid phase two-site enzyme immunoassay kit (Insulin ELISA 10–1113-01, Mercordia AB, Uppsala, Sweden) and s-FFA concentrations were determined using an enzymatic colorimetric method using a 96 well microplate (NEFA C, ACS-ACOD method, WAKO Chemicals GMbH, Germany). Serum triglycerides concentrations were analyzed with a multi-sample enzymatic assay (LabAssay™ Triglyceride 290–63,701, GPODAOS method, Wako Chemicals GmbH, Neuss, Germany). The quantitative determination of IL-1β and IL-6 in plasma were performed with enzyme immunoassays (Human IL-1β/IL-1F2 HSLB00D and Human IL-6 HS600B, respectively, R&D Systems, Abingdon, UK) and p-IL-18 with an enzyme immunoassay that was modified to eliminate that no dilution of plasma was performed prior to the analysis (Human IL-18 ELISA Kit 7620, MBL Medical & Biological Laboratories CO., Ltd., Nagoya, Japan). Enzyme immunoassay was also used to determine BDNF in plasma (ChemiKine BDNF Sandwich ELISA kit CYT306, Millipore Bioscience Research Reagents, USA and Canada), p-CRP (CRP ELISA Kit, Immunodiagnostik AG, Bensheim, Germany) and p-LBP (Human LBP ELISA Kit EKH3120, Nordic Biosite, Täby Sweden). Plasma GLP-2 and p-PYY (PYY (3–36) and PYY (1–36)) concentrations were determined with a competitive enzyme immunoassay (Human GLP-2 EIA YK141 and Human PYY EIA YK080, respectively, Yanaihara Institute Inc. Shizuoka, Japan). The quantitative determination of p-GLP-1 concentrations were performed using a highly sensitive ELISA enzyme-linked immunosorbent assay kit (GLP-1 (Active 7–36) ELISA 43-GP1HU-EO1 ALPCO Diagnostics, Salem, NH, USA). Plasma SCFA (acetate, propionate and butyrate) were analyzed using a GC method [33].

### Mood and cognitive tests

Measurements of mood and cognitive performance were performed after each of the two intervention periods.

#### Subjective mood assessment

The mood measurements were assed using the Swedish Core Affect Scale which included three self-reporting rating scales for evaluating valence (unpleasantness-pleasantness) and for activation (quietness-excitement), respectively [34]. The subjective mood ratings were registered using 100 mm VAS (described above in the section “Physiological test parameters”). The valence rating was assessed by obtaining a mean rating of three scales graded from *displeased-pleased, sad-glad* and *depressed-happy*, respectively, and the rating of activation was assessed by obtaining a mean rating of three scales graded from *sleepy-awake, passive-active* and *dull-peppy*, respectively [34].

#### Verbal working memory test

Each WM test session consisted of 48 short, declarative sentences, divided into 12 sets with 3, 4 or 5 sentences in each [25]. Half of the sentences in each session were semantically meaningful and the other half was nonsensical. The sentences were read one by one to the subject and they were instructed to answer immediately after each sentence if the sentence was ‘correct’ (i.e. semantically meaningful) or ‘incorrect’ (i.e. nonsensical). When a set of sentences were read to the subject, they should attempt to repeat the first noun in each sentence (in any order). The variable presented as results are the total number of nouns remembered. The WM tests were performed three times during the experimental days after each intervention period (at 90,120 and 160 min after commencing the breakfast). Each test took approximately 8 min to perform.

#### Selective attention test

The SA test was performed on a computer and measured the ability to maintain a prolonged attention, and to control and split the attention to the entire picture shown on the computer screen [35]. Similar to the WM test, the SA test requires storing and processing information simultaneously, however, the time for information storage was shorter and the pressure of time was higher. The test was made up of 96 pictures, each shown for 2 s on the screen. The subjects were instructed to compare each new picture that emerged on the screen with the previous one, and indicate with use of the keyboard spatial similarities between the pictures. The SA test was performed twice per experimental day (at 30 and 170 min post the standardized breakfast), and each test took approximately 10 min to perform. The test scores were based on the number of correct responses (in total 95 credits) and reaction time to give an answer, i.e. press a key button.

### Calculations and statistical methods

Data are expressed as means ± SEM. A trapezoid model was used to calculate the incremental area- and area under the curve (iAUC and AUC, respectively) for each subject and test meal. The iAUC was used in the statistical evaluation of results regarding blood glucose and insulin. AUC was used to display the appetite sensations. The peak concentrations, calculated for glucose and insulin, were calculated based on individual maximum postprandial increase from the baseline, using incremental values (iPeak). GraphPad Prism (version 7, GraphPad Software, San Diego, CA, USA) was used for graph plotting and calculation of areas.

Assessment of significant differences in test variables depending on intervention was performed with ANOVA (general linear model) using MINITAB Statistical Software (release 17; Minitab, Minitab Inc., State College, PA). The significance level was set at *P*-values < 0.05. In case of unevenly distributed residuals (tested with Anderson-Darling where *P* < 0.05 was considered unevenly distributed), Box Cox transformation was performed on the data prior to the ANOVA analysis. If a result on a test variable was missing for a subject after the test- or reference product, the test subject was excluded from the statistical calculations of that specific test variable. The differences between the interventions at different time points during the experimental day (‘Time’) were evaluated using a mixed model (PROC MIXED in SAS release 9.3; SAS Institute Inc., Cary, NC) with repeated measures and an autoregressive covariance structure for the test variables glucose, insulin, mood- and appetite parameters, and the WM- and SA tests. The relationships between mood and cognitive tests (WM- and SA tests), respectively, and glucose- and insulin responses, ISI_composite_ and HOMA-IR were investigated using Pearson correlation in Minitab statistical software (release 17; Minitab, Minitab Inc., State College, PA).

The composite insulin sensitivity index (ISI_composite_) was used to determine the insulin sensitivity. However, it was modified since the breakfast (WWB) included 50 g available carbohydrates instead of 75 g of glucose. ISI_composite_ was calculated by using the formula: 10000/square root of [FBG (mg/dl) × f-insulin (μU/ml) × mean glucose concentrations 0–120 min (mg·min/dl) × mean insulin concentrations 0–120 min (μU·min/ml)] [36]. The mean glucose- and insulin concentrations included in the formula, were based on blood glucose and s-insulin values measured every 30 min in the postprandial phase after the standardized breakfast. Homeostatic model assessment for insulin resistance (HOMA-IR) was calculated using formula: [f-glucose (mmol/L) × f-insulin (mU/L) / 22.5] [37]. The results in the WM-test were regarded as the primary outcome measure in this study. According to previous study including healthy middle aged subjects and a similar WM-test, 33 subjects would be sufficient to obtain 80% power (two tailed test) by assuming an effect size (Cohen’s d) of *d* = 0.50 to reach a significant effect. However, 43 subjects were recruited to the study by assuming a normal drop-out margin. The statistical calculations included 38 subjects.

## Results

### Study population

At the end of the trial, 38 subjects out of the 43 enrolled volunteers completed both intervention periods and were included in the statistical evaluation. The dropouts were due to difficulties to eat test product (*n* = 1), treatment with antibiotics (*n* = 2), or other personal reasons (n = 2). The physiological parameters included in the screening are presented in Table 2.

**Table 2:** The results of the physiological test parameters at screening^a^

Physiological parameters	Total (*n* = 38)	Women (*n* = 30)	Men (*n* = 8)
Age (years)	63.9 ± 5.5	63.7 ± 6.1	64.8 ± 2.1
BMI (kg/m^2^)	24.2 ± 2.5	24.0 ± 2.6	25.2 ± 2.2
Systolic BP (mmhg)	124.6 ± 19.8	121.8 ± 14.5	135.1 ± 32.4
Diastolic BP (mmhg)	82.4 ± 10.3	80.7 ± 8.3	88.8 ± 14.7
f-glucose (mmol/L)	5.7 ± 0.4	5.7 ± 0.4	5.6 ± 0.4

*BP* Blood pressure

^a^Data are presented as means ± SD

### Mood and cognition

#### Mood measurements - Swedish core affect scale

**Fig. 1:**
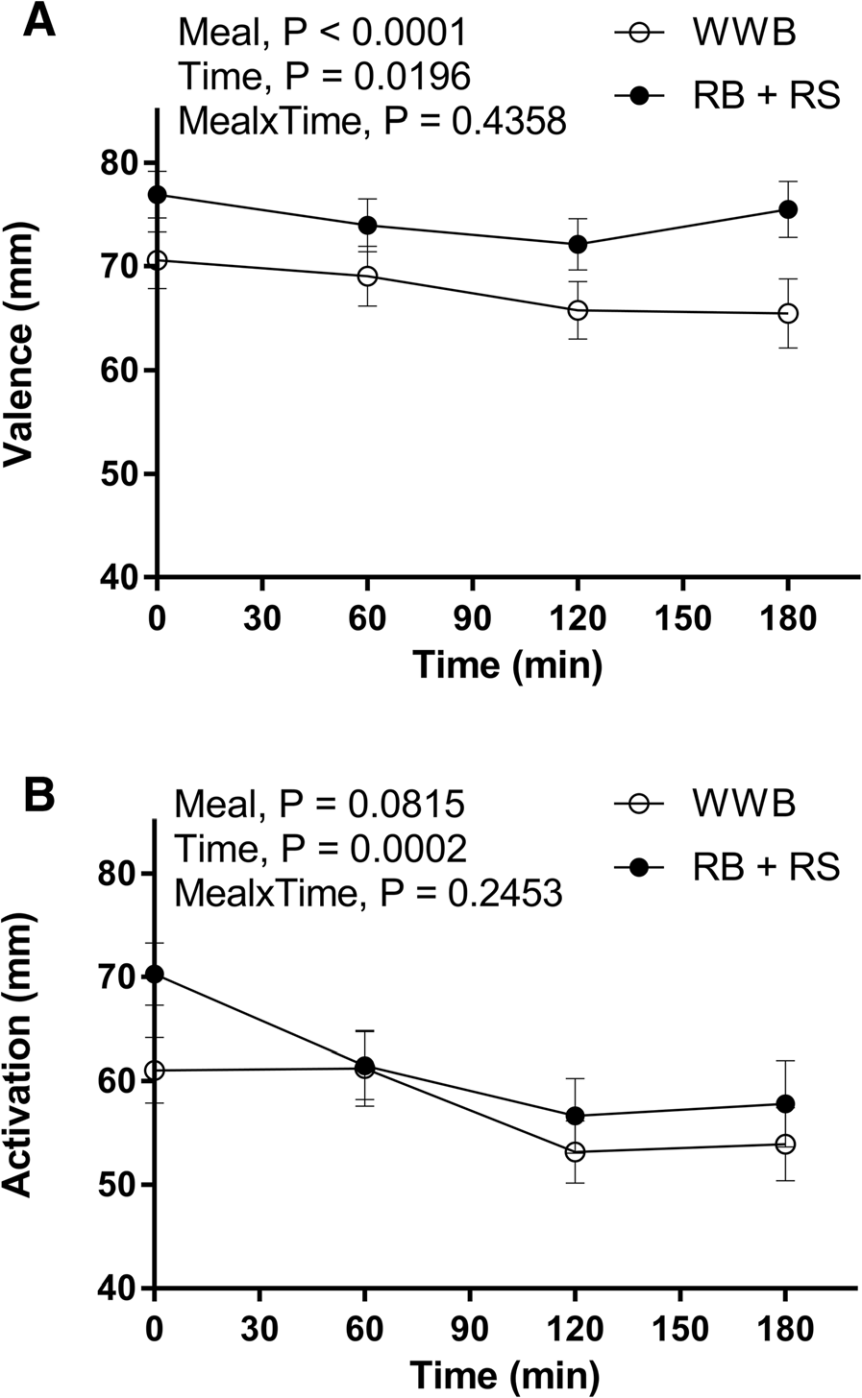
Subjective mood ratings (valence and activation) post the standardized breakfast following RB + RS2 or WWB intervention Valence ratings (**a**) include means of pleased, glad and happy. Activations ratings (**b**) include means of awake, active and peppy. Values are means ± SEM. Repeated measures; mixed model in SAS. RB + RS2, 1:1 ratio rye kernel- and flour based bread with added resistant starch (14%, dm); WWB, white wheat bread

The assessment of *valence* ratings revealed a significant increase after the RB + RS2 test product compared to the WWB, both at fasting (9%, *P* < 0.05) and following the standardized breakfast (mean 0–180 min, 10%, *P* < 0.001), i.e. 11–14 h post the RB + RS2 evening meal (Fig. 1 and Table 3). In addition, the RB + RS2 significantly increased the *activation* ratings at fasting (15%, *P* < 0.01) and during the experimental day (mean 0–180 min, 7%, *P* < 0.05), Fig. 1 and Table 3.

**Table 3:** Subjective mood ratings post the standardized breakfast, 11-14 h after RB + RS2 or WWB interventions, respectively^a^

Test variables	WWB	RB + RS2	%Δ^b^
Valence, fasting (mm)	70.6 ± 2.7	76.9 ± 2.3	9*
Valence, mean 0–180 min (mm)	67.8 ± 2.6	74.7 ± 2.2	10***
Activation, fasting (mm)	61.0 ± 3.2	70.3 ± 3.0	15*
Activation, mean 0–180 min (mm)	57.4 ± 2.5	61.6 ± 2.7	7*

*RB + RS2* 1:1 ratio rye kernel- and flour based bread with added resistant starch (14%, dm), *WWB* White wheat bread

*Different from WWB *P* < 0.05; **P < 0.01, ***P < 0.001 (ANOVA, GLM, Minitab)

^a^Data are presented as means ± SEM

^b^The percentage change was calculated as the difference from the WWB to the RB + RS2. *n* = 37

#### Cognitive tests (WM & SA tests)

The results in the cognitive tests *WM* and *SA* did not show any significant differences in correct response (CR) or reaction time, depending on test products (*P* > 0.05), Table 4.

**Table 4:** Performance results in the cognitive tests post the standardized breakfast after RB + RS2 or WWB, respectively^a^

Test variables	WWB	RB + RS2	%Δ^b^
WM test (max 48 CR)			
WM:1 (90 min)^c^	29.4 ± 0.8	30.0 ± 0.8	2
WM:2 (120 min)^d^	29.0 ± 0.7	29.7 ± 0.7	2
WM:3 (160 min)^d^	29.8 ± 0.8	29.5 ± 0.8	-1
WM:1–3 (mean)^d^	29.3 ± 0.7	29.6 ± 0.7	1
SA-test			
The complete SA tests (max 95 CR)			
SA:1 (30 min)^e^	69.8 ± 3.3	71.2 ± 3.3	2
SA:2 (170 min)^f^	71.9 ± 3.2	72.2 ± 3.2	0
SA:1–2 (mean)^e^	70.1 ± 3.2	70.9 ± 3.1	1
The last half of the SA tests (max 48 CR)			
SA:1 (30 min)^e^	34.9 ± 1.6	36.0 ± 1.7	3
SA:2 (170 min)^f^	35.8 ± 1.5	35.3 ± 1.6	-1
SA:1–2 (mean)^e^	35.0 ± 1.5	35.3 ± 1.6	1
The complete SA tests, reaction time (ms)			
SA:1 (30 min)^e^	1256 ± 26	1248 ± 28	-1
SA:2 (170 min)^f^	1189 ± 25	1176 ± 29	-1
SA:1–2 (mean)^e^	1233 ± 25	1218 ± 26	-1
The last half of the SA tests reaction time (ms)			
SA:1 (30 min)^e^	1263 ± 30	1225 ± 29	−3
SA:2 (170 min)^f^	1188 ± 27	1173 ± 29	-1
SA:1–2 (mean)^e^	1238 ± 28	1203 ± 26	-3

*CR* Correct response, *RB + RS2* 1:1 ratio rye kernel- and flour based bread with added resistant starch (14%, dm), *SA* Selective attention, *WM* Working memory, *WWB* White wheat bread

^a^Data are presented as means ± SEM

^b^The percentage change was calculated as the difference from the WWB to the RB + RS2

^c^*n* = 36

^d^*n* = 38

^e^*n* = 37

^f^*n* = 35

### Glucose and insulin

**Fig. 2:**
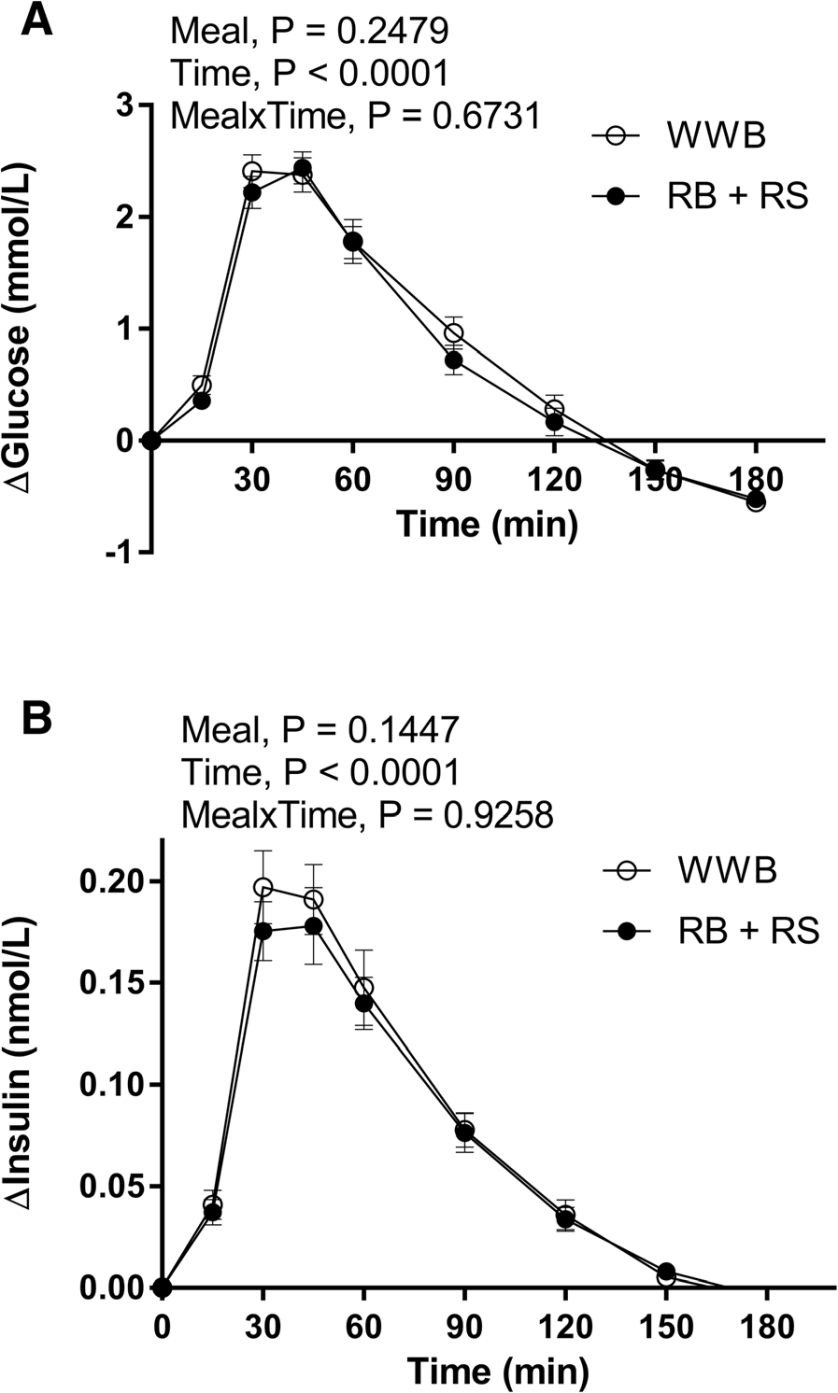
Incremental glucose and insulin responses post the standardized breakfast following RB + RS2 or WWB interventions Changes in mean incremental concentrations from fasting concentrations of: **a** blood glucose and **a** serum insulin, 11–14 h after RB + RS2 or WWB intake. Values are means ± SEM. Repeated measures; mixed model in SAS. RB + RS2, 1:1 ratio rye kernel- and flour based bread with added resistant starch (14%, dm); WWB, white wheat bread

No significant differences in fasting concentration of glucose and insulin were observed, compared to WWB. The incremental blood glucose response (iAUC) 0–30 min post breakfast was significantly decreased after RB + RS2 (− 14%, *P* < 0.05) compared to WWB, Fig. 2 and Table 5. RB + RS2 significantly lowered the incremental insulin peak value (iPeak, − 15%, *P* < 0.01) and increased the insulin sensitivity (ISI_composite_) (11%, P < 0.05), compared to WWB. No significant differences in postprandial incremental insulin response (iAUC) or incremental glucose peak value (iPeak) were observed (Fig. 2 and Table 5).

**Table 5:** Glucose and insulin measures post the standardized breakfast, 11-13 h after RB + RS2 or WWB intake^a^

Test variables	WWB	RB + RS2	%Δ^b^
b-Glucose, fasting (mmol/L)	5.7 ± 0.1	5.7 ± 0.1	0
b-Glucose, iAUC 0–30 min (mmol·min/L)	26.1 ± 2.0	22.5 ± 1.9	−14*
b-Glucose, iAUC 0–120 min (mmol·min/L)	155.5 ± 11.5	143.6 ± 8.6	−8
b-Glucose, iPeak (mmol/L)	2.8 ± 0.1	2.7 ± 0.1	−3
s-insulin, fasting (nmol/L)	0.036 ± 0.002	0.033 ± 0.002	−10
s-insulin, iAUC 0–30 min (nmol·min/L)	2.1 ± 0.2	1.9 ± 0.2	−10
s-insulin, iAUC 0–120 min (nmol·min/L)	12.6 ± 1.1	11.8 ± 1.0	− 7
s-insulin, iPeak (nmol/L)	0.24 ± 0.02	0.20 ± 0.02	−15**
Insulin Sensitivity Index (ISI_composite_)	9.1 ± 0.5	10.1 ± 0.6	11*
HOMA-IR	1.5 ± 0.1	1.4 ± 0.1	−9

*n* = 38

*AUC* Area under curve, *b* Whole blood, *HOMA-IR* Homeostatic model assessment of insulin resistance, *i* Incremental, *ISI*_*composite*_ Insulin sensitivity index, *Peak* Mean including individual peak value, *RB + RS2* 1:1 ratio rye kernel- and flour based bread with added resistant starch (14%, dm), *s* Serum, *WWB* White wheat bread

*Different from WWB *P* < 0.05; **P < 0.01 (ANOVA, GLM, Minitab)

^a^Data are presented as means ± SEM

^b^The percentage change was calculated as the difference from the WWB to the RB + RS2

### Gut hormones

There was a significant increase in f-plasma concentration of PYY (9%, *P* < 0.05) and GLP-2 (10%, P < 0.01), 11 h post intake of RB + RS2 compared to WWB, Table 6. No significant differences in GLP-1 fasting concentrations were observed depending on treatment (Table 6).

**Table 6:** Fasting concentrations of gut hormones, SCFA and breath H_2_, 11 h post RB + RS2 or WWB intake^a^

Test variables	WWB	RB + RS2	%Δ^b^
p-PYY, fasting (ng/mL)^c^	0.44 ± 0.06	0.47 ± 0.07	9*
p-GLP-1, fasting (pg/mL)^c^	16.2 ± 2.4	17.4 ± 2.9	7
p-GLP-2, fasting (ng/mL)^c^	8.5 ± 0.9	9.4 ± 1.2	10**
breath H_2_, fasting (ppm)^d^	18 ± 3	42 ± 18	139***

*GLP* Glucagon-like peptide, *H*_*2*_ Breath hydrogen excretion, *p* Plasma, *PYY* Peptide YY, *RB + RS2* 1:1 ratio rye kernel- and flour based bread with added resistant starch (14%, dm), *WWB* White wheat bread

*Different from WWB *P* < 0.05; **P < 0.01, ***P < 0.001 (ANOVA, GLM, Minitab)

^a^Data are presented as means ± SEM

^b^The percentage change was calculated as the difference from the WWB to the RB + RS2

^c^*n* = 37

^d^*n* = 38

### SCFA and breath hydrogen

**Fig. 3:**
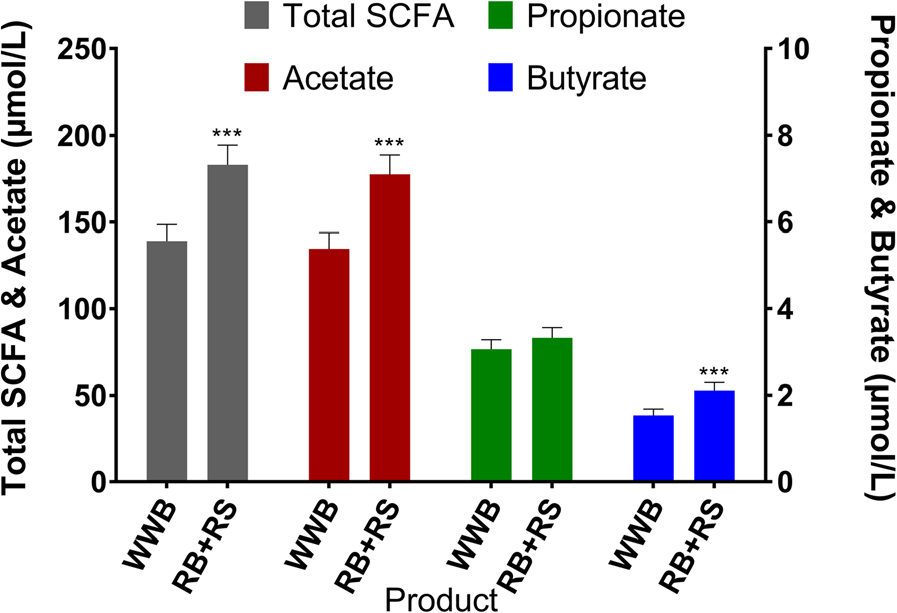
Fasting concentrations of total and individual SCFA following RB + RS2 or WWB intervention Values are means ± SEM. RB + RS2, 1:1 ratio rye kernel- and flour based bread with added resistant starch (14%, dm); SCFA, short chain fatty acids; WWB, white wheat bread

The total f-SCFA plasma concentrations, including acetate, propionate and butyrate, were significantly increased 11 h after intake of RB + RS2 evening meal (32%, *P* < 0.001), compared to WWB (Fig. 3 and Table 6). In addition, there was a significant increase of the individual SCFA acetate (32%, P < 0.001) and butyrate (37%, P < 0.001) after the RB + RS2 test product. No significant differences depending on intervention were observed regarding propionate (*P* = 0.126). The RB + RS2 also significantly increased the breath H_2_ excretion at fasting (139%, *P* < 0.001) (Table 6).

### Inflammatory markers, blood lipids and BDNF

The RB + RS2 evening meal decreased the inflammatory marker IL-1β concentrations significantly at fasting (− 11%, P < 0.05), compared to WWB, Table 7. Concerning the other inflammatory markers (CRP, IL-6, IL-18 and LBP) and blood lipids (FFA and triglycerides), no significant differences were observed depending on intervention (*P* > 0.05, Table 7). Neither were there any differences with respect to BDNF.

**Table 7:** Fasting concentrations of inflammatory markers, blood lipids and BDNF, 11 h after RB + RS2 or WWB intake^a^

Test variables	WWB	RB + RS2	%Δ^b^
p-CRP, fasting (ng/mL)^c^	1890 ± 580	1650 ± 320	−13
p-IL-1β, fasting (pg/mL)^d^	0.085 ± 0.011	0.076 ± 0.011	−11*
p-IL-6, fasting (pg/mL)^c^	2.0 ± 0.2	1.9 ± 0.2	−4
p-IL-18, fasting (pg/mL)^e^	177 ± 12	176 ± 12	0
p-LBP, fasting (pg/mL)^e^	93.0 ± 6.6	98.2 ± 6.7	6
s-FFA, fasting (mmol/L)^d^	0.31 ± 0.02	0.35 ± 0.03	12
s-Triglycerides, fasting (mg/dL)^c^	109 ± 7	116 ± 7	6
p-BDNF, fasting (ng/mL)^e^	0.59 ± 0.04	0.56 ± 0.04	−5

*BDNF* Brain-derived neurotrophic factor, *CRP* C-reactive protein, *FFA* Free fatty acids, *IL* Interleukin, *LPB* Lipopolysaccharide binding protein, *RB + RS2* 1:1 ratio rye kernel- and flour based bread with added resistant starch (14%, dm), *WWB* White wheat bread

*Different from WWB *P* < 0.05 (ANOVA, GLM, Minitab)

^a^Data are presented as means ± SEM

^b^The percentage change was calculated as the difference from the WWB to the RB + RS2

^c^*n* = 37

^d^*n* = 36

^e^*n* = 38

### Subjective appetite ratings

No significant differences in subjective appetite sensations (satiety, hunger or desire to eat) were observed at fasting or in the postprandial period during the experimental day depending on preceding intervention, (P > 0.05, Table 8).

**Table 8:** Subjective appetite ratings post the standardized breakfast, 11-14 h after RB + RS2 or WWB intervention, respectively^a^

Test variables	WWB	RB + RS2	%Δ^b^
Satiety, fasting (mm)	41.7 ± 4.0	43.8 ± 4.1	5
Satiety, AUC 0-180 min (mm·min)	9700 ± 380	10,060 ± 430	4
Hunger, fasting (mm)	48.3 ± 3.7	48.8 ± 3.8	1
Hunger, AUC 0-180 min (mm·min)	7570 ± 470	7000 ± 470	−7
Desire to eat, fasting (mm)	56.6 ± 3.7	54.6 ± 3.5	−3
Desire to eat, AUC 0-180 min (mm·min)	8420 ± 530	7850 ± 530	−7

*n* = 38

*AUC* Area under curve, *RB + RS2* 1:1 ratio rye kernel- and flour based bread with added resistant starch (14%, dm), *WWB* White wheat bread

^a^Data are presented as means ± SEM

^b^The percentage change was calculated as the difference from the WWB to the RB + RS2

### Correlations

#### Relationships between markers of glucose regulation (glucose, insulin, and insulin sensitivity) and mood variables

In the morning post WWB intervention, a significant negative correlation was observed between insulin concentrations (iAUC 0–120 min) and the later phase of valence (120–180 min) (*r* = − 0.339, *P* = 0.04).

Furthermore, when dividing valence into the different mood parameters, the glucose and insulin responses mainly correlated with the ratings of happiness (depressed-happy). Thus, a significant negative correlation between insulin iAUC 0–120 min response and happiness at the time point 120 min was noticed after the WWB intervention (*r* = − 0.351, *P* = 0.033) and a similar trend was shown after the RB + RS2 intervention (*r* = − 0.312, *P* = 0.06).

In addition, a trend towards an inverse correlation was observed between glucose response (iAUC 0–120 min) and happiness (time point 120 min) post the WWB intervention (*r* = − 0.299, *P* = 0.073). On the other hand, a positive correlation was detected between fasting valence ratings and both FBG (*r* = 0.399, *P* = 0.044) and HOMA-IR (*r* = 0.364, *P* = 0.027) after WWB.

#### Relationships between markers of glucose regulation (glucose, insulin, and insulin sensitivity) and performance in cognitive tests

The insulin sensitivity index (ISI_composite_) was positively correlated with the WM tests after both interventions. Thus, a significant correlation following RB + RS2 was shown between ISI_composite_ and the performance in the total WM test (WM:1–3, *r* = 0.328, *p* = 0.044). Furthermore, a significant correlation following WWB was observed between ISI_composite_ and the performance in the WM test at 150 min after the standardized breakfast (WM:3, *r* = 0.332, *p* = 0.041), Table 9. Additionally, negative correlations were observed between performance in WM tests and insulin iAUC (0–150 min), both following intake of RB + RS2 (WM:2 & WM:3, *P* < 0.05), and following WWB (WM:3, *P* < 0.05), Table 10. In addition, post RB + RS2 intervention an inverse correlation appeared between glucose iAUC 0–150 min and the second SA test performed at 170 min after commencing the standardized breakfast (*r* = − 0.337, *P* = 0.047).

**Table 9:** Correlations between ISI_composite_ and WM tests performance post the standardized breakfast after RB + RS2 or WWB

	ISI_composite_			
		WM:1–3 (mean)	WM:2	WM:3
Treatment: WWB	*r*	0.294	0.215	0.332
	*P*	0.073	0.194	0.041
Treatment: RB + RS2	*r*	0.328	0.438	0.296
	*P*	0.044	0.006	0.071

*n* = 38

*ISI*_*composite*_ Insulin sensitivity index, *RB + RS2* 1:1 ratio rye kernel and flour based bread with added resistant starch (14%, dm), *WM* Working memory, *WWB* White wheat bread

**Table 10:** Correlations between insulin response and WM tests performance post standardized breakfast after RB + RS2 or WWB

	Insulin iAUC (0–150 min)			
		WM:1–3 (mean)	WM:2	WM:3
Treatment: WWB	*r*	−0,312	−0,256	−0,361
	*P*	0,056	0,12	0,026
Treatment: RB + RS2	*r*	−0,285	−0,373	− 0,326
	*P*	0,083	0,021	0,046

*n* = 38

*iAUC* Incremental area under the curve, *RB + RS2* 1:1 ratio rye kernel and flour based bread with added resistant starch (14%, dm), *WM* Working memory, *WWB* White wheat bread

## Discussion

This study undertook to investigate the effects of short-term intervention with WG rye on cognitive functions, mood and cardiometabolic risk markers, and to investigate possible relationships thereof. One of the main findings concerns the increased ratings of mood parameters, both valance and activation, in the morning after the RB + RS2 intervention compared to after the WWB intervention. Furthermore, the RB + RS2 intervention increased insulin sensitivity, decreased fasting concentrations of IL-1β and induced higher fasting plasma concentrations of gut hormones PYY and GLP-2, compared to the WWB intervention. Additionally, the fasting concentrations of acetate and butyrate, and the breath H_2_ excretion were significantly increased when preceded by the RB + RS2 intervention, indicating increased gut fermentation activities. Other important findings were the significant inverse relationships between parameters of glucose regulation with both mood parameters and performance in cognitive tests. Hence, the postprandial insulin response was negatively correlated with both feelings of happiness and performance in the WM-tests after intake of RB + RS2 and WWB, respectively. In addition, a trend towards negative correlations following WWB was observed between insulin iAUC (0–150 min) and the total WM test; WM:1–3, *P* = 0.056. A trend towards a negative correlation was also observed between the SA test performed at 170 min post breakfast and insulin iAUC 0–150 min after the RB + RS2 evening meal (*r* = − 0.308, *P* = 0.072).

Cardiometabolic risk factors such as chronic hyperglycemia, oxidative stress, increased low grade inflammation and insulin resistance are all probable factors involved in the underlying mechanisms whereby T2D increase the risk of cognitive impairments and depressive symptoms [38]. Thus, measures to lower these risk factors must thus be taken, not only in prevention of cardiometabolic complications, but also to prevent a concomitant impairment of cognitive decline and depressive symptom. Furthermore, relationships exists between cardiometabolic risk variables and cognitive functions also in healthy individuals [24, 26]. Consequently, it was shown in an apparently healthy cohort of 40 subjects (aged 49–70 years), divided in two groups depending on having glucose tolerance below or above the median of the studied group, that performance on WM- and SA tests was superior in the 20 subjects that have higher glucose tolerance compared to the 20 with lower glucose tolerance [39]. Similarly it was observed that a progressively worse glucose regulation predicted poorer performance on measures of WM and executive function-in nondiabetic middle aged and older subjects (49–70 years). Together these results suggest a progressive decline in cognitive functions and deteriorations prior to glucoregulatory impairment reaches levels consistent with a T2D diagnosis. Thus, both metabolic and cognitive impairments are progressive processes with no sharp cut off values, which is important to be taken into consideration in prevention strategies. As described above and in the Introduction section, improvements in cardiometabolic risk markers and cognitive performance have previously been reported in healthy subjects after intervention with certain foods. Improvements have been seen on glucose regulation e.g. after short term intervention (1–3 days) with barley kernel based meals [10, 11, 40] and rye kernels [9], thus indicating preventive potential with respect to metabolic diseases and related cognitive decline. A glucose profile characterized by less oscillating glucose concentrations has proven beneficial with respect to prevent acute postprandial insulin resistance [41–43], and it has also been demonstrated in healthy subjects that cognitive functions may be enhanced acutely in the postprandial phase after intake of foods resulting in a metabolically favorable glucose excursion, characterized by a low peak increment but higher postprandial blood glucose concentrations in the late postprandial period, compared with a high glycaemic index food [26, 44]. One suggested contributing factor to the impaired cognitive functions is a decreased insulin sensitivity that may occur acutely after a high-GI meal, which may affect brain functions, possibly together with decreased glucose supply to the brain [26, 39, 44]. The results from previous studies thus show that cognitive functions may be related to cardiometabolic variables also in apparently healthy subjects, and that foods and diets may affect metabolic risk variables and cognitive functions also in a short term.

The presently described study is the first study to investigate effects of short-term intervention with WG rye-based products on cognitive functions in terms of WM and SA, and mood variables in terms of valence and activation parameters. No significant differences depending on intervention were observed regarding cognitive performance, however, novel findings were observed concerning increased subjective mood ratings after the RB + RS2 evening meal. Both the mood parameters valence and activation were significantly improved during the experimental day, i.e. 11–14 h post intake of RB + RS2, indicating that the subjects were more prone towards feeling pleased, glad and happy (valance), and awake, active and peppy (activation) after RB + RS2, compared to WWB. The impact of WG intervention on mood has not previously been widely studied but acute positive effects on mood with increased ratings of happiness and alertness in the postprandial period has been reported after intake of a cereal bar (1.1 g DF per portion) [45]. Concomitantly with the improved mood ratings in the current study, the RB + RS2 intervention also resulted in a significant increase in insulin sensitivity post the following breakfast.

Diabetic patients have increased risk of depression and reversely, those who are depressed have increased risk of diabetes [46]. Interestingly, correlations between mood parameters and postprandial insulin concentrations were observed in the present study. Thus, the results indicated that a higher rating of being ‘happy’ (the mood rating ‘depressed’ on the opposite side of the same VAS-scale) was related to a lower iAUC insulin response, especially in the later part of the experimental day, suggesting relationships between insulin sensitivity and mood also in healthy individuals.

In the present study no significant effects were observed on cognitive performance depending on intervention. It can be argued whether the duration of the intervention, i.e. 3 days, was long enough to observe semi-acute effects of RB + RS2 compared to WWB on cognitive functions in the healthy middle-aged subjects. However, we observed an inverse correlation between glucose tolerance (iAUC 0–150 min) and performance in the SA test at 170 min (correct responses), which is in agreement with previous studies showing relationships between cognitive functions and glucose regulation [26, 39, 44]. In addition, the observed negative correlation in the current study between postprandial insulin concentrations (iAUC 0–150 min) and the performance in the WM test at 120 and 150 min, as well as the positive correlations between increased insulin sensitivity and total WM performance, are important novel findings and can add to the understanding of underlying mechanisms between T2D and increased risk of impaired cognitive functions and mood. In particular the observation indicates a role of foods that facilitate glucose regulation in maintenance of good mood. The correlations between cognitive functions and insulin in this study is in accordance with previous observation in T2D patients with Alzheimer’s disease showing improvements in cognitive functions when improving the insulin sensitivity [47]. Since the metabolic syndrome and T2D are associated with cognitive decline [48], it would potentially be possible to prevent, and also to some extent reverse, the cognitive impairment related to metabolic dysfunction. In this respect diet strategies for prevention is important and measures to increase intake of WG rye kernel/flour mixture are probably advantageous.

In the present study, we also observed a significant reduction in IL-1β concentrations concomitant with increased concentrations of GLP-2 at fasting after RB + RS2. IL-1β is a pro-inflammatory cytokine and increased concentrations of IL-1β have been observed in obese individuals, and animals with diet-induced obesity [49]. Further IL-1β is associated with impaired insulin secretion [49]. On the contrary, higher GLP-2 concentrations have been reported to be induced in healthy subjects approximately 11 h after intake of non-digestible carbohydrates in e.g. brown beans and barley kernel products [11, 50]. GLP-2 is involved in the maintenance of gut barrier functions and possesses anti-inflammatory properties by obstructing influx of lipopolysaccharides (LPS) from the gut lumen. According to Cani and coworkers (2009), feeding mice prebiotics (oligofructose) caused increased production of GLP-2 together with lowering of inflammatory markers such as LPS, IL-1 (α and β) and IL-6, suggesting an anti-inflammatory role of GLP-2 due to enhancement of the gut barrier function [51]. In addition, GLP-2 appears to have anti-inflammatory properties also in the brain, i.e. reducing the response of IL-1β, IL-6 and TNF-α in mouse microglial cell line as observed following pre-treatment of the cells with GLP-2 prior to LPS stimulation [52]. However, in the present study only IL-1β was significantly different depending on intervention product and further investigations are needed to understand the link between fermentation of DF in rye, GLP-2 and inflammatory tonus.

The effects on cardiometabolic risk markers after intake of the RB + RS2 product in a middle aged cohort is in accordance with results with a similar rye products (including 85% rye kernels (dm)) evaluated in the same time perspective in young healthy adults [12]. One exception between the present study and the latter is the somewhat less prominent reduction of postprandial blood glucose and insulin iAUC 0–120 min post the standardized breakfast in the presently described study. Thus, in the current study intake of RB + RS2 in the evening improved the early (0–30 min) postprandial glucose- and insulin responses to the standardized breakfast. The study in young adults did not include cognitive tests, and it is possible that the demanding cognitive tests, starting at 30 min post commencing the breakfast, may have interfered with the postprandial glucose concentrations [53]. In contrast to the previous study in young adults, the results in the present study displayed a trend towards increased f-FFA levels post RB + RS2 (*P* = 0.072) compared to WWB. Moreover, in the current study a trend was noticed towards increased TG post RB + RS2 intervention (*P* = 0.065). Elevated FFA concentrations have shown to impair insulin signaling [54]. In the present study however, we observed significantly increased insulin sensitivity after the RB + RS2. Consequently, further studies are needed to investigate the effects of WG rye on TG and FFA in middle-age subjects.

In accordance with the previous study in young healthy subjects (25.3 ± 3.9 years), the rye-based product RB + RS2 increased the satiety-inducing gut hormone PYY at fasting 11 h after intake, without significant effects on subjective appetite ratings [12]. The results regarding PYY are in accordance with other studies [55]. PYY and GLP-1 are produced by L-cells in response to food intake and have displayed anti-obesogenic potential. Accordingly, both PYY and GLP-1 administered via peripheral infusions reduced food intake in humans [14]. In addition, these gut hormones have been ascribed anti-diabetic properties, e.g. GLP-1 is regarded as an incretin hormone and thus have shown to enhance insulin secretion in response to food. Today GLP-1 analogues are included in drug therapy to patients with diabetes [15]. PYY have been suggested as an early predictor of T2D, based on observations where postprandial PYY concentrations are blunted in individuals that are genetically susceptible for T2D [56]. Furthermore, animal studies have shown that PYY has anti-depressant effects [27] and GLP-1 has shown to contribute to improve cognitive functions such as memory and learning [28]. Consequently, upregulation of these gut hormones may positively affect several metabolic, cognitive, and mood aspects. However, despite a slight non-significant increase in GLP-1 (7%), no significant differences in f-GLP-1 levels were observed depending on intervention. It has been shown in young adults that both GLP-1 and PYY are stimulated after a rye kernel based meal [9] through gut fermentation of DF. Accordingly, the increased concentrations of SCFA and H_2_ in the present study, indicate that the gut fermentation activity was higher after the RB + RS2 evening meal. Increased formation of acetate and butyrate has also previously been observed after intake of WG rye kernel based evening meals [9]. SCFA are suggested to influence colonic L-cells, resulting in stimulation of GLP-1 and PYY release [13], which is in accordance with the significantly increased concentrations of SCFA and PYY after RB + RS2 in this study. Altogether the results from this study suggest that foods rich in specific indigestible carbohydrates, e.g. WG rye based foods, may have the potential to beneficially affect brain functions via colonic fermentation mechanisms, including the “gut-brain axis”. However, evaluations regarding effects on metabolic and cognitive variables should be performed also in longer term interventions to validate solid beneficial effects. Evaluations should preferably be performed also in subject groups with impaired metabolic regulation to investigate possible health benefit that may contribute to prevent further metabolic deterioration, and concomitant decline in cognitive functions and mood parameters.

No increase in BDNF concentrations were observed after RB + RS2 despite increased colonic fermentation activity and formation of SCFA and gut hormones. Thus, the results in this study could not support previous observations of increased BDNF in healthy young adults after intervention with WG rye kernel-based bread [21]. The reasons for the inconsistency in results regarding effects of rye bread on BDNF concentrations, and also on GLP-1, compared with previous studies, have to be further investigated, but one reason could be the differences between the compositions of the rye bread products. In the previous study the bread was made from 85% whole rye kernels (dry basis) while the bread in the presently described study instead was made of 43% rye kernels, 43% WG rye flour, and 14% added RS type 2. The results thus suggest that intact structure of the rye kernels would be more favorable with respect to stimulate an increase in BDNF and GLP-1, and supplementation with RS type 2 could not substitute for the botanically encapsulated RS in the whole kernels. An additional explanation to the discrepancy in the results could be the difference in age between the two study groups; middle aged subjects in the current study and young adults (25.3 ± 3.9 years) in the previous study.

## Conclusions

This is to the best of our knowledge the first study to display improvements on mood ratings post intake of WG rye (kernel/flour mixture) supplemented with RS, along with inverse correlations between mood ratings and insulin response, in middle-aged subjects 11–14 h after intake. In addition, the results revealed that short-term RB + RS2 intervention have the potential to improve insulin sensitivity, and increase plasma levels of gut hormones involved in glucose and appetite regulation (PYY). Moreover, levels of GLP-2, involved in gut barrier function, were increased, whereas the inflammatory marker IL-1β was decreased. The increase in fermentation metabolites suggest that the beneficial effects are mediated through gut microbial fermentation of the DF in RB + RS2. Altogether, the results support a view of WG rye products with a kernel and flour mixture having anti-diabetic potential, useful in the prevention of T2D and with the possibility of ameliorating also mood.

## Abbreviations

AUC: Area under curve
b-: Blood-
BDNF: Brain-derived neurotrophic factor
BMI: Body mass index
BP: Blood pressure
CR: Correct response
CRP: C-reactive protein
CVD: Cardiovascular disease
DF: Dietary fiber
dm: Dry matter
f-: Fasting-
FFA: Free fatty acids
GLP: Glucagon-like peptide
H_2_: Breath hydrogen
HAMRS2: High-amylose maize resistant starch type 2
HOMA-IR: Homeostatic model assessment of insulin resistance
iAUC: Incremental area under curve
IL: Interleukin
iPeak: Incremental peak
ISI_composite_: Insulin sensitivity index
LPB: Lipopolysaccharide binding protein
LPS: Lipopolysaccharide
NSP: Non-starch polysaccharides
p-: Plasma-
PYY: Peptide YY
RB + RS2: 1:1 ratio rye kernel- and flour based bread with added resistant starch (14%, dm)
RS: Resistant starch
SA: Selective attention
SCFA: Short chain fatty acids
SD: Standard deviation
SEM: Standard error mean
T2D: Type 2 diabetes
VAS: Visual analogue scale
WG: Whole grain
WM: Working memory
WWB: White wheat bread
